# Population Morphometry of *Conger myriaster* (Anguilliformes: Congridae) along the Coast of China: Implications for Population Structure and Fishery Management

**DOI:** 10.3390/ani14132007

**Published:** 2024-07-07

**Authors:** Peiyi Xiao, Tianyan Yang

**Affiliations:** Fishery College, Zhejiang Ocean University, Zhoushan 316022, China; xiaopeiyi@zjou.edu.cn

**Keywords:** *Conger myriaster*, morphology, population structure, fishery management, China coast

## Abstract

**Simple Summary:**

Understanding the composition of a population in terms of its demographic characteristics, spatial distribution, and social organization within a population is of great importance for policymakers, researchers, and organizations to make informed decisions and develop effective strategies. To better understand the population structure and fishery biological background of *Conger myriaster*, an economically important marine fish, five meristic counts and seventeen morphological measurements from seven different geographical populations along coastal China were analyzed by traditional morphometry method. The univariate and multivariate statistical analysis results revealed a geographical population structure of *C. myriaster*. In the face of undergoing long-term fishing pressure and massive catching efforts, biogeography-based fishery strategies for *C. myriaster* should be employed in the coastal waters of China.

**Abstract:**

In this study, the traditional morphometry method was applied to analyze the standardized measurements together with the meristic counts so as to provide supplementary information for fishery biology, population assessment, and fishery resources protection of *C. myriaster*. The results of one-way analysis of variance (ANOVA) showed that the greatest divergence was observed between the Dalian and Qingdao populations, whereas the smallest difference was found between the Lianyungang and Zhoushan populations. Statistical difference in tail length (*TAL*) was detected between all populations. The morphological traits with high *C.D* values were mostly related to body weight (*BW*), confirming greater potential variations of these weight-related traits. Principal component analysis (PCA) extracted 7 principal components (PCs) with eigenvalues greater than 1, and the cumulative contribution rate was 72.790%. The results of cluster analysis, together with the PCA and DFA, supported separating the populations into three groups linked with their geographic distribution and their specific environment localization. Considering the particularity of the natural environment of the Bohai Sea and the sophisticated oceanic circulations of the Shandong Peninsula, the relationships of *C. myriaster* populations in the northwest Pacific Ocean along the China coast were closely related to their geographical distributions and oceanic circulations.

## 1. Introduction

Phenotypic plasticity is an important biological mechanism for adapting to perpetual environmental fluctuations, which refers to the degree of change in bio-phenotype relative to environmental variations and has more producing capability than one alternative form of morphology, physiological state, and/or behavior [1,2]. The most common manifestation of phenotypic plasticity is population differentiation from distinct regions or habitats, which can be caused by the intervention of several factors, such as genetic variation, geographic isolation (habitat fragmentation), historical change, human impact, and ecological dynamism (inter and intra-species relationships) [3,4,5,6,7]. As the most basic content of biological research, fish morphometry has been widely used in stock assessment, germplasm conservation, and genetic breeding nowadays, which lays the foundation for further research on comparative biology, population genetics, phylogeny, and adaptive evolution [8,9,10,11]. Morphometric measurements have been widely used to identify differences between fish populations. Turki-Missaoui et al. [12] used the traditional morphological method to detect obvious differentiation between *Sander lucioperca* populations in relatively similar environments, although they were derived from a single gene pool. Myoung and Kim [13] conducted a multivariate morphometric analysis of *Konosirus punctatus* and clarified two morphotypes on the Korean coasts. Turan et al. [14] studied the genetic and morphological variation of *Pomatomus saltatrix* based on morphometric and meristic analyses. The results detected three morphologically differentiated groups and the highest reclassification rate for the eastern Black Sea and northeastern Mediterranean Sea samples.

*Conger myriaster* (Brevoort, 1856) [15], commonly known as Whitespotted conger, is an economically valuable marine fish species widely distributed in the northwest Pacific, from the southern East China Sea to the coasts of Japan and Korean Peninsula [16], and occupies a significant proportion in import and export trades of fishery resources in Asian countries. As a warm-water demersal fish, *C. myriaster* mainly inhabits muddy and gravelly bottoms in coastal waters with depths of 5~50 m and possesses a critical position in the nearshore food web and marine ecosystem [17]. Recent studies showed that fishery resources of *C. myriaster* in the Yellow Sea and the East China Sea had undergone over-fishing pressure to a certain extent [18,19]. Meanwhile, environmental changes, together with human activities, are also assumed to induce resource fluctuations substantially, which can cause profound impacts on the population structure and distribution of this species. Most literature on *C. myriaster* mainly focused on early life history, feeding ecology, reproductive biology, and population genetics [20,21,22,23,24,25]. As for morphologic references on *C. myriaster*, only the path analysis of morphological traits on body weight [26] and the comparative morphological study of only two populations in coastal China were available [27].

Traditional morphometry was regarded as the most basic method of population identification and taxonomic classification before the emergence of barcoding and molecular ecology because morphological traits can reflect external resemblance and partial inherent genetic characteristics [3]. Understanding the population structure and biological background of target catches should be an essential prerequisite for designing and establishing fishery management policies [28]. Therefore, it was particularly important to carry out more detailed morphological studies of distinct *C. myriaster* populations so as to provide basic data for sustainable exploitation and utilization of germplasm resources. In the present study, the morphological characteristics of *C. myriaster* from seven different geographic locations along the coastal waters of China were comprehensively compared and analyzed for the first time; besides, the intraspecific morphological variations and differentiation patterns were investigated. The results will provide some supplementary information for fishery biology research of *C. myriaster*, as well as the basic data for the population assessment and protection of the fishery resources.

## 2. Materials and Methods 

### 2.1. Material

#### Samples Collection and Morphometric Measurement

**Figure 1:**
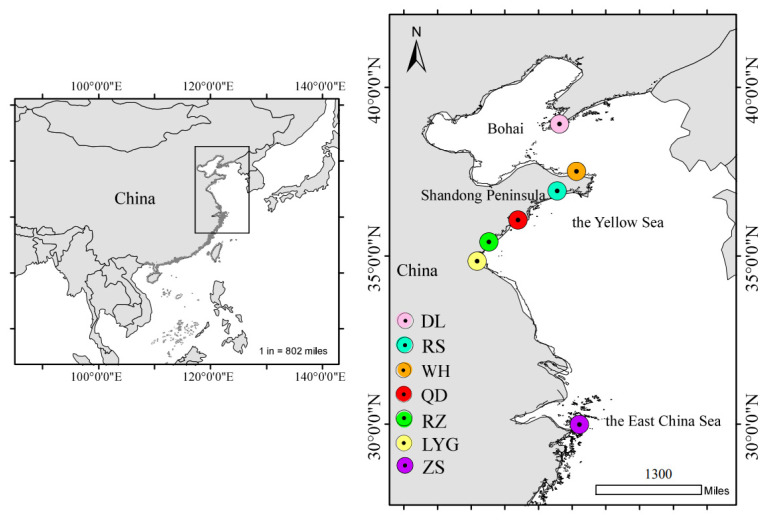
Sampling map of *C. myriaster* populations. (DL, WH, RS, QD, RZ, LYG, and ZS stand for the Dalian population, Weihai population, Rushan population, Qingdao population, Rizhao population, Lianyungang population, and Zhoushan population, respectively).

A total of 218 *C. myriaster* individuals were collected by the commercial trawling from 7 different geographical sites along coastal China: Dalian (DL), Weihai (WH), Rushan (RS), Qingdao (QD), Rizhao (RZ), Lianyungang (LYG) and Zhoushan (ZS) from August 2022 to May 2023 (Table 1; Figure 1).

**Figure 2:**
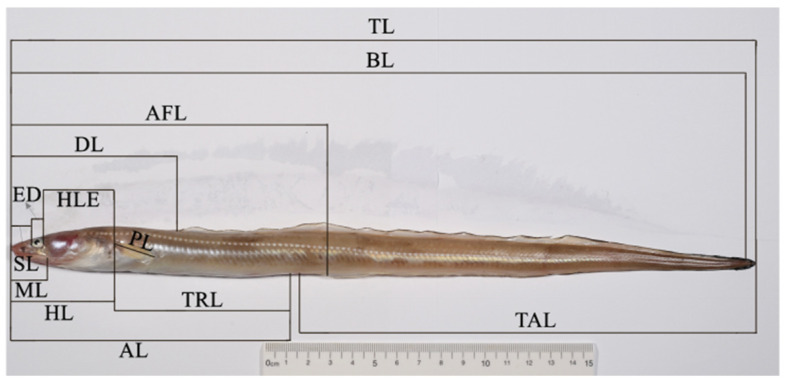
Analyzed morphological measurements of *C. myriaster* in this study. (The measurements *HB*, *EI*, *BD*, and *BB* are not marked in the figure due to the angle of view. Detailed information of analyzed meristic counts and morphological measurements are shown in Table S1).

Iced fresh fishes were quickly transported to the Fishery Ecology and Biodiversity Laboratory (FEBL) of Zhejiang Ocean University of China. Then, experimenters immediately cleaned the samples in running water, drained them with absorbent paper, and placed them on a flat platform. Each individual was separately labeled with a specific code for identification, and the body weight (*BW*) was measured by using an electronic balance accurate to 0.01 g. The morphological analysis data comprised 5 vertebral counts and 17 morphological measurements (Table S1; Figure 2). Morphological measurements were always recorded from the left side of the fish body using a ruler and vernier caliper, with 0.1 cm and 0.01 mm precisions, respectively. Vertebral counts were carried out by removing the back muscles to expose the vertebrae. To ensure the reliability of this study, any sample that showed extensive damage or dysplasia was eliminated, meanwhile, all operations were performed by the same person.

### 2.2. Data Analysis

In order to eliminate variations resulting from allometric growth, morphometric measurement data were initially logarithmically converted. The standardization was conducted by an allometric method, as suggested by Elliott et al. (1995) [29]:

*M*_S_ = *M*_0_ (*L*_S_/*L*_0_) *^b^* (1)

*b* = log_10_*^M^*^0^/log_10_*^L^*^0^ (2)

In Formulas (1) and (2), *M*_S_ is the standardized measurement data, *M*_0_ is the original measurement data, *L*_S_ is the overall mean of the original total length (*TL*) for all fish samples in each population, and *L*_0_ is the original total length (*TL*).

The above-gathered data were made a preliminary arrangement in Microsoft Excel 2021, and then the variation coefficient (*C.D*) was calculated according to Mayr’s 75% identification and division rules [30]:

*C.D* = |*M*_1_ − *M*_2_|/(*S*_1_ + *S*_2_) (3)

In Formula (3), *M*_1_ and *M*_2_ represent the average values of measurement data in two populations *S*_1_ and *S*_2_ represent the standard deviations of measurement data in two populations.

Univariate and multivariate statistical analysis of the standardized data was performed by using one-way analysis of variance (ANOVA), hierarchical cluster analysis (HCA), discriminant function analyses (DFA), and principal component analysis (PCA), all of which were completed in SPSS 27.0 [31]. A univariate ANOVA was done to test whether there was any statistically significant difference for each morphometric character between pairwise populations. In ANOVA, Fisher’s least significant difference (LSD) test was used to analyze measurement data with homogeneity of variances [32], while Tamhane’s T2 test was used to analyze measurement data without homogeneity of variances [33]. Cluster analysis was applied to determine the closely related populations and draw the dendrogram based on the Euclidean distances. DFA and PCA were used to examine any phenotypic differences between populations. A stepwise discriminant procedure can reduce the number of variables in order to meet the requirement of reduced discriminant function analysis [34]. The discriminant equations were constructed, and the ability to differentiate populations was evaluated through original-validation tests [35,36,37]. PCA can also select morphometric data to extract several independent variables for population differentiation [38]. In order to more intuitively observe the differences among populations, OriginPro 2021 software was used to draw scatter plots based on the first and second principal components (PCs).

## 3. Results

### 3.1. The One-Way Analysis of Variance

One-way ANOVA results of both meristic counts and morphological measurements among seven *C. myriaster* populations are shown in Table S2. Twenty-two morphological indexes were statistically significant (*p* < 0.05) except for *DAV*/*TV*, *BL*, and *AL*. There were 17 morphological indexes with significant differences between the Dalian and Qingdao populations, but only 3 indexes between the Lianyungang and Zhoushan populations were significantly different. The *C.D* values of meristic counts and morphological measurements between two *C. myriaster* populations obtained in this study were all below 1.28, indicating that the diversity difference mainly originated from inter-populations. At the same time, the larger *C.D* values of *AFV*, *AFL*, *AL*, *TRL*, and *EI* implied greater variations in the longitudinal body axis of this species (Table S3). Considering the large *C.D* values of tail length (*TAL*) and no significant difference in the mean body length, the variations of fish bodies were affected by the seasonal predation and nutrient structure presumably.

### 3.2. The Principal Component Analysis

A total of 7 PCs with eigenvalues greater than 1 were extracted, and the cumulative contribution rate was 72.790%. The contribution rate of the PC1 accounted for 18.839% with the larger load values of *DV*, *AFV*, *ML*, *ED*, *EI*, and *BB*. The PC2 accounted for 16.120%, and *AV*, *AFV*, *AV*/*TV*, *AFV*/*TV*, *AFL*, and *TRL* attained the higher load values (Table 2). These indexes mainly reflected the head features and the vertebral count changes. Moreover, *ML*, *AFL*, *TRL*, and *BB* were also associated with the predation habit and barrel-shaped body structure of *C. myriaster*.

**Figure 3:**
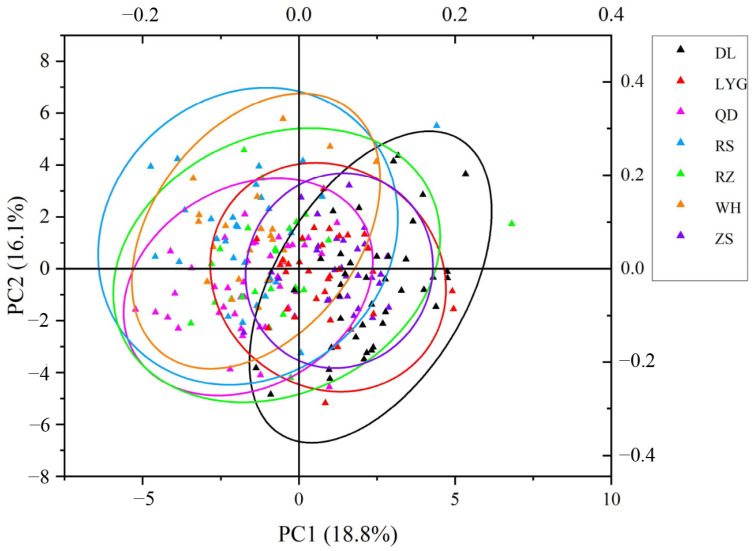
PCA scatter plot based on the first and second principal components. (DL, WH, RS, QD, RZ, LYG, and ZS stand for the Dalian population, Weihai population, Rushan population, Qingdao population, Rizhao population, Lianyungang population, and Zhoushan population, respectively).

A scatter plot based on PCA showed that individuals in the Rushan and Rizhao populations were more dispersed than others, and both of them occupied broader areas. Individuals from the Lianyungang population almost encircled those of the Zhoushan population, and both of them possessed a considerable percentage of common area with the Rizhao population. All populations gathered with each other, and the overlapping area between the Weihai and Dalian populations was minimal (Figure 3).

### 3.3. The Cluster Analysis

**Figure 4:**
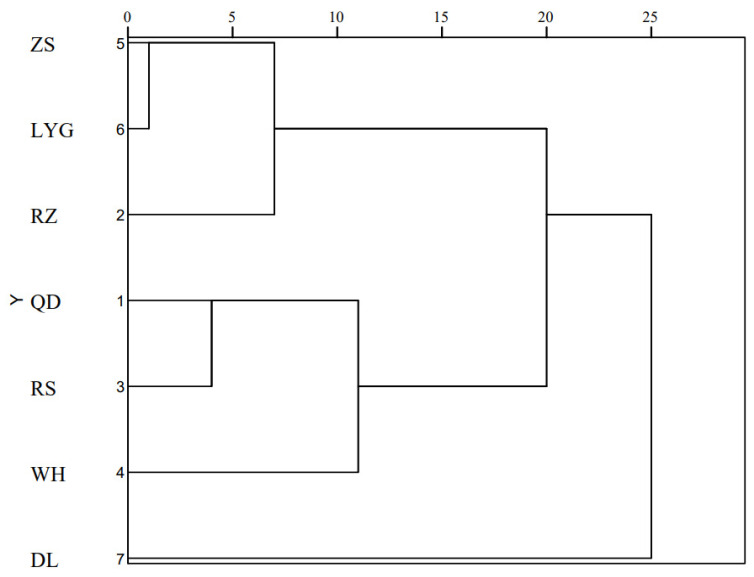
The dendrogram based on Euclidean distances of *C. myriaster* populations. (DL, WH, RS, QD, RZ, LYG, and ZS stand for the Dalian population, Weihai population, Rushan population, Qingdao population, Rizhao population, Lianyungang population, and Zhoushan population, respectively).

Cluster analysis was carried out to display the similarity among populations. According to the Euclidean distances of 7 *C. myriaster* populations (Table S4), the relationship between Zhoushan and Lianyungang populations was the closest, with an Euclidean distance of only 3.261. While the Euclidean distance between Weihai and Zhoushan populations reached 17.710, showing quite a few differences. The dendrogram showed that the Dalian population had a distant relationship with other populations, and formed an independent branch. Individuals from the remaining six geographic locations belonging to the Yellow Sea and the East China Sea were generally co-clustered into two major branches, respectively, suggesting obvious differences between the two sea areas (Figure 4).

### 3.4. The Discriminant Function Analysis

**Figure 5:**
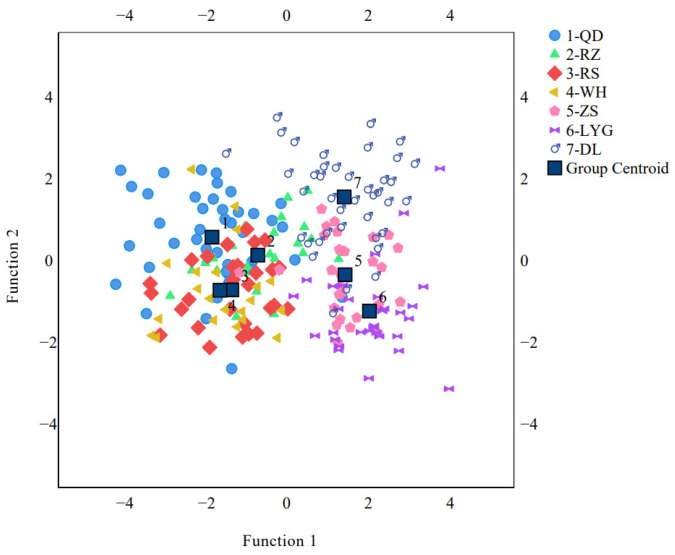
Discriminant analysis plot with 25 morphometric variables of *C. myriaster* populations. (DL, WH, RS, QD, RZ, LYG, and ZS stand for the Dalian population, Weihai population, Rushan population, Qingdao population, Rizhao population, Lianyungang population, and Zhoushan population, respectively).

The stepwise discriminant analysis was conducted according to the correlations between the independent variables and the dependent variables. In this study, a total of 9 characteristics (*AFV*, *AV*/*TV*, *BL*, *AFL*, *DL*, *TRL*, *EI*, *HLE*, and *PL*) were retained in the stepwise discriminant analysis, and 4 typical discriminant functions (DFs) with significant differences were obtained. The cumulative contribution rate of DF1 and DF2 reached 86.8%, which meant the functions included the most meaningful morphometric characteristics. Scatter plots based on DFA mainly divided the *C. myriaster* populations into three groups (Figure 5), which were almost consistent with their distribution of sea areas.

The Dalian population was clearly distinguished from the other populations, forming an independent group with slight overlap, and the group centroid was the furthest from the other populations in either the vertical or horizontal axis. The populations in the Yellow Sea did not distribute in regularity. The Qingdao, Rushan, and Weihai populations clustered together and became another independent group. Despite being the most geographically distant, the Zhoushan (the northern East China Sea) and Lianyungang (the southern Yellow Sea) populations exhibited small differences and constituted the third group. Only the Rizhao population was a special one, which was slightly superposed with the latter two groups. These latter two groups were partially overlapped, and the distances of group centroids on the horizontal axis were greater than those on the vertical axis. Among all the populations, the group centroid of Qingdao and Lianyungang populations had the furthest distance on the horizontal axis, and the group centroid of Dalian and Lianyungang populations had the furthest distance on the vertical axis.

According to the results of discriminant analysis, the discriminant functions of each population were constructed as follows:

*Y*_QD_ = 10.122*AFV* + 2644.927*AV*/*TV* + 54.614*BL* + 0.522*AFL* + 5.098*DL* + 4.408*TRL* + 0.326*EI* + 2.318*HLE* + 1.623*PL* − 1858.731

*Y*_RZ_ = 11.757*AFV* + 2541.173*AV*/*TV* + 53.962*BL* + 0.405*AFL* + 5.264*DL* + 4.323*TRL* − 0.159*EI* + 2.430*HLE* + 1.765*PL* − 1865.746

*Y*_RS_ = 11.624*AFV* + 2580.945*AV*/*TV* + 53.880*BL* + 0.567*AFL* + 5.147*DL* + 4.352*TRL* − 0.270*EI* + 1.775*HLE* + 1.775*PL* − 1866.559

*Y*_WH_ = 11.550*AFV* + 2574.959*AV*/*TV* + 54.523*BL* + 0.646*AFL* + 5.253*DL* + 4.237*TRL* − 0.896*EI* + 2.360*HLE* + 1.542*PL* − 1894.879

*Y*_ZS_ = 13.101*AFV* + 2477.486*AV*/*TV* + 52.806*BL* + 0.625*AFL* + 4.790*DL* + 3.789*TRL* + 0.375*EI* + 2.411*HLE* + 2.480*PL* − 1845.895

*Y*_LYG_ = 13.587*AFV* + 2382.272*AV*/*TV* + 52.600*BL* + 0.704*AFL* + 4.847*DL* + 3.677*TRL*−0.166*EI* + 2.037*HLE* + 3.294*PL* − 1840.668

*Y*_DL_ = 12.175*AFV* + 2606.883*AV*/*TV* + 52.974*BL* + 0.268*AFL* + 5.403*DL* + 3.769*TRL* + 0.452*EI* + 3.084*HLE* + 2.312*PL* − 1853.890

The discriminant accuracies are shown in Table S5, in which the discriminant accuracy of the Dalian population is the highest (72.50%). The DFA demonstrates that about 59.63% of inspected individuals are correctly classified into the original population on average.

## 4. Discussion

A sufficient degree of habitat isolation may lead to significant intraspecific phenotypic and genetic differentiation [39]. Reproductive isolation of fish populations can occur in different spawning areas due to hydrological characteristics that reduce or even prevent cross-regional migration [40,41]. In general, fishes are more susceptible to environmentally-induced morphological variation, allowing them to exhibit higher variations within and between populations than other vertebrates [42]. These morphological changes can be used as a basis for the distinction and management of different populations [37].

In this presently reported study, the multivariate analysis was employed to reflect the external morphology differences of *C. myriaster* in various areas of coastal China. The results of one-way ANOVA showed that the differences between the Dalian and Qingdao populations were the largest, and individuals in the Dalian population (the Bohai Sea) also held more significant differences from others. Previous studies revealed that the bottom fishery biological communities in the Yellow and Bohai Sea had been under serious disturbance in recent decades [43]. The environmental medley of factors might lead to great differences between these two populations located in different sea areas mentioned above. There were no significant differences in body length (*BL*) and length before anal (*AL*), but significant differences in tail length (*TAL*) were detected among all populations in ANOVA. Furthermore, the morphological traits with high variation coefficient values were mostly related to body weight (*BW*), which might be due to the special long-strip shape of eels. Tail length (*TAL*) reflecting the swimming ability of fishes can further affect their predatory ability and make these weight-related traits have greater potential variation [44].

PCA conclusions mainly manifested that the cumulative contribution rate of seven PCs’ eigenvalue greater than 1 was less than 85%. Subsequent related studies could increase the traits analyzed or adopt other morphological analysis methods to conduct in-depth research according to actual situations [45]. Zou et al. (2020) [46] used mitochondrial control region sequences to assess the genetic diversity, population differentiation, and demographic history of *C. myriaster*. It was revealed that no clear genetic subdivision was found among populations, which is consistent with the conclusions of Ishikawa [21]. However, our results of multivariate analysis were generally consistent with the actual geographical distributions of each population. The cluster analysis together with the plots of PCA and DFA separated the populations into three groups: the north group (DL), the central group (WH, QD, and RS), and the south group (RZ, LYG, and ZS). A similar population structure of *Scomberomorus niphonius* from the eight spawning grounds along the Chinese coast was also detected, according to a morphometric truss network method [47]. It was speculated that the unique geographical location of the Bohai Sea, the only semi-enclosed inland sea, limited intraspecific communication and resulted in the specialization of morphological traits. Consequently, the Dalian population occupied an isolated space and was relatively farther away across the straits. Coincidentally, strong morphometric and meristic differentiation resulted from the closed geographic structure, and environmental discreteness was also observed in horse mackerel (*Trachurus mediterraneus*) from the Marmara Sea by Turan [48]. The Shandong Peninsula is the largest peninsula in China, with a coastline length of more than 3000 km and accounting for about 1/6 of the total of China. The ambient conditions determined the composition and spatial distribution of macrobenthos in offshore areas of the Shandong Peninsula. The bottom temperature is a principal environmental element that is closely related to the macrozoobenthic community structure. The coastal currents, water masses, and continental shelf front interfere with the distribution of bottom temperature in the Yellow Sea [49].

As a bottom-dwelling eel with metamorphosis development, the habitat and migratory behaviors of *C. myriaster* are both affected by marine currents and the surroundings [50,51]. The literature evidenced that *C. myriaster* spawned along the Kyushu-Palau Ridge in the western North Pacific, even if it remained controversial [22,23]. Takai [52] suggested that the spawning grounds of this species were at the edge of the continental shelf in the East China Sea with the pattern of “a single spawning ground for a single population”. Instead, Mochioka et al. [20] believed that Whitespotted congers living in East Asian waters had multiple composite spawning populations. Until recent years, researchers investigated the breeding areas and migration patterns of *C. myriaster* by utilizing the age-structured otolith chemistry profiles and confirmed again that *C. myriaster* in coastal China seas came from the same spawning area located in the western North Pacific [53]. The larvae and juveniles may be passively transported from their spawning area to the East China Sea together by the North Equatorial Current and the Kuroshio Current before being separated by the Yellow Sea Warm Current to different habitats during the subsequent life stages. Morphometric characters are susceptible to environmental influences throughout life. In addition, phenotypic variations between populations are easily influenced by local circumstances, often associated with the geographic regions that the species occupies throughout its ranges [54]. To name but a few examples, three independent stocks of *Harpadon nehereus* along the Indian coast were detected by the multiple morphological comparisons, and future policy regulations should consider collaboration among the maritime state governments since fishery on the west coast of India was replenished by a single stock [55]. The morphological variation of *Liparis tanakae* sampled from three localities surrounding the Korean peninsula was analyzed, and the canonical discriminant analysis clearly revealed three groups that separated according to locality [56]. Besides, the feeding relationships of marine organisms and their trophic positions in the food web are not always static but are affected by seasonal temperatures, showing a dynamic change process [57]. Therefore, the different sampling times are also likely to have a certain impact on fish morphological variation and differentiation. By comprehensively considering the multiple factors, we inferred that the population structure and spatial pattern of *C. myriaster* on the coast of China were possibly influenced directly or indirectly by the unique environmental elements, sloshing bottom fishery biological communities, coastal currents, water masses, and continental shelf front.

## 5. Conclusions

Summarizing the morphological multivariate data, we found that the relationships of *C. myriaster* populations in the northwest Pacific Ocean along China’s coast were closely related to their geographical locations, where the population structure was most likely influenced by sophisticated oceanic circulations together with the various other environmental factors to a certain extent. As recent studies showed that marine resources in the Yellow and East China Seas were undergoing long-term fishing pressure and massive catching efforts, the demarcation of jurisdictional boundaries for *C. myriaster* must be fully considered, and biogeography-based fishery management units (FMUs) should be established to ensure sustainable utilization of *C. myriaster*.

## Figures and Tables

**Table 1:** Sampling information of *C. myriaster*.

Sampling Sites	Sea Regions	Geographical Coordinates	Sampling Time	Sample Size	Total Length (TL)	
					Range (cm)	Mean ± SD (cm)
Dalian (DL)	The Bohai Sea	38°55′ N; 121°37′ E	2023-05	40	33.7–51.7	45.7 ± 3.9
Weihai (WH)	The Yellow Sea	37°31′ N; 122°7′ E	2022-09	21	28.9–36.6	32.7 ± 2.2
Rushan (RS)		36°56′ N; 121°32′ E	2022-10	29	27.6–45.7	35.0 ± 4.3
Qingdao (QD)		36°4′ N; 120°23′ E	2022-09	41	30.6–39.2	35.0 ± 2.1
Rizhao (RZ)		35°25′ N; 119°32′ E	2022-08	25	33.9–48.4	38.2 ± 3.3
Lianyungang (LYG)		34°36′ N; 119°13′ E	2023-04	32	30.6–50.4	39.1 ± 3.7
Zhoushan (ZS)	The East China Sea	29°59′ N; 122°12′ E	2022-10	30	30.6–45.1	40.8 ± 3.9

**Table 2:** Load values and contribution rates of seven principal component factors.

Index	Principal Component (PC)						
	1	2	3	4	5	6	7
*TV*	0.393	−0.149	−0.547	−0.104	0.640	−0.096	−0.091
*DV*	0.596	0.255	−0.568	0.428	−0.007	0.050	0.047
*AV*	0.482	0.641	−0.417	−0.131	0.159	0.029	−0.005
*AFV*	0.593	0.561	−0.371	−0.268	0.220	−0.080	−0.011
*DAV*	0.115	0.420	0.209	−0.779	0.240	−0.158	0.014
*DV*/*TV*	0.538	0.326	−0.456	0.497	−0.200	0.081	0.080
*AV*/*TV*	0.183	0.807	0.021	−0.049	−0.372	0.111	0.069
*AFV*/*TV*	0.357	0.786	0.037	−0.221	−0.293	−0.010	0.064
*DAV*/*TV*	−0.095	0.473	0.480	−0.673	−0.106	−0.096	0.060
*BL*	0.153	−0.331	−0.095	−0.125	0.075	0.176	0.716
*TAL*	0.293	−0.309	0.131	−0.254	0.274	0.339	0.006
*AFL*	−0.406	0.593	0.417	0.273	0.322	0.132	0.060
*AL*	−0.121	−0.058	0.060	0.205	−0.240	−0.606	0.324
*DL*	−0.089	0.456	0.363	0.572	0.174	0.202	−0.128
*TRL*	−0.492	0.594	0.127	0.181	0.037	0.299	0.049
*HL*	0.190	0.244	0.673	0.432	0.279	−0.167	−0.001
*HB*	0.436	−0.282	0.081	−0.089	−0.352	0.414	−0.141
*SL*	0.438	−0.129	0.178	−0.082	−0.220	−0.212	−0.520
*ML*	0.584	−0.025	0.184	0.242	−0.147	−0.384	0.081
*ED*	0.658	−0.134	0.296	−0.047	0.139	−0.165	0.057
*EI*	0.606	−0.192	0.217	0.050	0.043	0.126	−0.088
*HLE*	0.525	0.016	0.550	0.307	0.224	−0.098	0.068
*BD*	0.450	−0.162	0.433	−0.080	0.012	0.213	0.109
*BB*	0.578	−0.205	0.296	−0.095	−0.190	0.303	0.124
*PL*	0.483	−0.204	0.291	0.009	−0.035	−0.018	0.019
Eigenvalues	4.710	4.030	3.090	2.573	1.471	1.314	1.010
Contribution ratio (%)	18.839	16.120	12.361	10.291	5.884	5.256	4.038
Cumulative contribution ratio (%)	18.839	34.960	47.320	57.612	63.495	68.751	72.790

## Data Availability

The data used in this study are available from the corresponding author upon reasonable request.

## Supplementary Materials

The following supporting information can be downloaded at: https://www.mdpi.com/article/10.3390/ani14132007/s1, Table S1: Analyzed meristic counts and morphological measurements of *C. myriaster* in this study; Table S2: One-way analysis of variance (ANOVA) results of seven *C. myriaster* populations; Table S3: The variation coefficient (*C.D*) values for each trait of pairwise populations of *C. myriaster*; Table S4: Euclidean distances of seven *C. myriaster* populations; Table S5: The accuracy of discriminant function analyses in each population.
