# Supplementary material for: Population Morphometry of Conger myriaster (Anguilliformes: Congridae) along the Coast of China: Implications for Population Structure and Fishery Management
Source: Animals (Basel). 2024 Jul 7;14(13):2007. doi: 10.3390/ani14132007 (PMC11240619; doi:10.3390/ani14132007)
Supplement: Supplementary file 1 [file animals-14-02007-s001.zip › animals-3026998-supplementary.pdf]

Table S1. Analyzed meristic counts and morphological measurements of *C. myriaster* in this study.

**Type of characteristics**	**Characteristic descriptions**	**Abbreviation**
Morphological measurements	Total length	*TL*
	Body length	*BL*
	Tail length	*TAL*
	Length before anal fin	*AFL*
	Length before anal	*AL*
	Length before dorsal fin	*DL*
	Trunk length	*TRL*
	Head length	*HL*
	Head breadth	*HB*
	Snout length	*SL*
	Length of mouth crack	*ML*
	Eye diameter	*ED*
	Eye distance	*EI*
	Head length behind the eye	*HLE*
	Body depth	*BD*
	Body breadth	*BB*
	Length of pectoral fin	*PL*
Meristic counts	Number of total vertebrae	*TV*
	Number of vertebrae before the dorsal fin	*DV*
	Number of vertebrae before the anal	*AV*
	Number of vertebrae before the anal fin	*AFV*
	Number of vertebrae between dorsal fin-anal fin	*DAV*

Table S2. One-way analysis of variance (ANOVA) results of seven *C. myriaster* populations. (*DV*/*TV*, *AV*/*TV*, *AFV*/*TV* and *DAV*/*TV* are the ratios of meristic counts, respectively. The same superscripts mean nonsignificant difference (P>0.05), while different superscripts mean significant difference (P<0.05).

**Traits**	**DL**	**WH**	**RS**	**QD**	**RZ**	**LYG**	**ZS**	***P***	**Total**
	**Mean ± SE**	**Mean ± SE**	**Mean ± SE**	**Mean ± SE**	**Mean ±SE**	**Mean ± SE**	**Mean ± SE**		**Mean ± SE**
TV	143.38±0.466^{bc}	142.57±1.027^{c}	141.83±0.452^{c}	139.15±0.735^{d}	142.56±0.448^{c}	144.53±0.359^{ab}	145.03±0.499^{a}	0.000	142.60±0.257
DV	13.38±0.171^{a}	12.81±0.245^{bc}	12.62±0.283^{c}	11.80±0.168^{d}	12.72±0.255^{c}	12.91±0.164^{abc}	13.27±0.143^{ab}	0.000	12.77±0.083
AV	39.53±0.237^{ab}	39.52±0.235^{ab}	39.52±0.220^{ab}	38.07±0.208^{c}	39.12±0.240^{b}	39.16±0.186^{b}	39.77±0.141^{a}	0.000	39.18±0.089
AFV	42.60±0.253^{bc}	42.19±0.214^{c}	42.31±0.233^{c}	40.56±0.210^{d}	42.24±0.247^{c}	43.06±0.185^{ab}	43.30±0.180^{a}	0.000	42.26±0.104
DAV	29.15±0.236^{b}	29.38±0.176^{ab}	29.69±0.233^{ab}	29.10±0.308^{b}	29.52±0.265^{ab}	30.16±0.201^{a}	30.03±0.182^{a}	0.008	29.55±0.097
DV/*TV*	0.09±0.001^{a}	0.09±0.002^{ab}	0.09±0.002^{b}	0.08±0.001^{c}	0.09±0.002^{b}	0.09±0.001^{b}	0.09±0.001^{ab}	0.000	0.09±0.001
AV/*TV*	0.28±0.002^{ab}	0.28±0.003^{a}	0.28±0.002^{a}	0.27±0.001^{ab}	0.27±0.002^{ab}	0.27±0.001^{b}	0.27±0.001^{ab}	0.019	0.27±0.001
AFV/*TV*	0.30±0.002^{a}	0.30±0.002^{ab}	0.30±0.002^{a}	0.29±0.001^{b}	0.30±0.002^{ab}	0.30±0.001^{a}	0.30±0.002^{a}	0.018	0.30±0.001
DAV/*TV*	0.20±0.001^{a}	0.21±0.002^{a}	0.21±0.002^{a}	0.21±0.002^{a}	0.21±0.002^{a}	0.21±0.001^{a}	0.21±0.002^{a}	0.190	0.21±0.001
BL	31.23±0.105^{a}	31.22±0.140^{a}	30.91±0.268^{a}	31.46±0.121^{a}	31.32±0.103^{a}	31.07±0.148^{a}	31.12±0.184^{a}	0.232	31.20±0.060
TAL	22.33±0.163^{a}	21.48±0.088^{c}	21.54±0.313^{bc}	21.90±0.095a^{bc}	21.94±0.085^{ab}	22.00±0.070^{a}	22.17±0.060^{a}	0.001	21.95±0.060
AFL	126.27±1.761^{e}	140.89±1.157^{a}	136.47±1.347^{b}	134.92±0.705^{bc}	132.16±1.232^{cd}	131.90±0.973^{cd}	130.40±0.901^{d}	0.000	132.73±0.546
AL	133.03±13.835^{a}	131.60±1.008^{a}	128.12±1.244^{a}	127.92±0.625^{a}	123.57±1.196^{a}	121.99±0.859^{a}	120.67±0.823^{a}	0.812	126.87±2.549
DL	62.25±0.667^{b}	64.75±0.641^{a}	62.79±0.498^{b}	62.31±0.601^{b}	62.44±0.617^{b}	60.44±0.529^{c}	59.93±0.396^{c}	0.000	62.01±0.239
TRL	78.46±0.609^{d}	85.75±0.634^{a}	85.68±0.651^{a}	84.31±0.522^{ab}	83.44±0.847^{b}	80.17±0.519^{c}	80.22±0.380^{c}	0.000	82.29±0.291
HL	45.33±0.445^{ab}	46.10±0.497^{a}	44.01±0.373^{c}	44.81±0.311^{bc}	43.77±0.514^{c}	44.56±0.412^{bc}	43.94±0.271^{c}	0.002	44.65±0.158
HB	18.14±0.201^{a}	15.54±0.296^{d}	17.02±0.273^{c}	17.41±0.219^{bc}	17.31±0.364^{bc}	17.74±0.318^{ab}	17.88±0.202^{ab}	0.000	17.41±0.109
SL	12.60±0.166^{a}	11.69±0.153^{bcd}	11.37±0.143^{d}	11.52±0.230^{cd}	11.35±0.244^{d}	11.93±0.129^{bc}	12.01±0.105^{b}	0.000	11.82±0.074
ML	18.35±0.179^{a}	16.94±0.169^{bc}	16.05±0.255^{d}	16.56±0.243^{cd}	16.72±0.385^{c}	17.43±0.209^{b}	17.02±0.138^{bc}	0.000	17.07±0.100
ED	7.44±0.087^{a}	6.60±0.119^{b}	6.41±0.127^{b}	6.65±0.121^{b}	6.57±0.274^{b}	7.18±0.085^{a}	7.15±0.079^{a}	0.000	6.90±0.055
EI	12.38±0.170^{a}	9.77±0.267^{d}	10.33±0.136^{cd}	11.23±0.300^{b}	10.90±0.232^{bc}	11.04±0.210^{b}	11.84±0.138^{a}	0.000	11.20±0.099

HLE	29.47±0.284^{a}	28.06±0.265^{b}	26.79±0.275^{c}	27.91±0.197^{b}	27.82±0.366^{b}	28.28±0.304^{b}	28.35±0.142^{b}	0.000	28.17±0.113
BD	18.87±0.234^{a}	17.77±0.435^{b}	16.77±0.290^{c}	18.07±0.300^{ab}	17.66±0.441^{bc}	18.02±0.330^{ab}	18.24±0.239^{ab}	0.000	17.98±0.125
BB	19.10±0.263^{a}	16.53±0.487^{d}	16.72±0.358^{d}	17.71±0.284^{bc}	17.33±0.329^{cd}	18.17±0.352^{bc}	18.32±0.265^{ab}	0.000	17.83±0.134
PL	21.54±0.248^{b}	19.26±0.327^{c}	19.39±0.226^{c}	20.10±0.253^{c}	19.73±0.265^{c}	23.23±0.581^{a}	21.17±0.189^{b}	0.000	20.75±0.150

Table S3. The variation coefficient (*C.D*) values for each trait of pairwise populations of *C. myriaster*.

**Traits**	**DL/ WH**	**DL/ RS**	**DL/ QD**	**DL/ RZ**	**DL/ LYG**	**DL/ ZS**	**WH/ RS**	**WH/ QD**	**WH/ RZ**	**WH/ LYG**	**RS/ QD**	**RS/ RZ**	**RS/ LYG**	**RS/ ZS**	**QD/ RZ**	**QD/ LYG**	**QD/ ZS**	**RZ/ LYG**	**RZ/ ZS**	**LYG/ ZS**
TV	0.105	0.287	0.552	0.157	0.232	0.292	0.104	0.364	0.002	0.291	0.376	0.157	0.605	0.620	0.492	0.799	0.791	0.462	0.497	0.105
DV	0.257	0.290	0.728	0.278	0.234	0.058	0.071	0.456	0.037	0.047	0.314	0.036	0.117	0.280	0.389	0.549	0.785	0.084	0.265	0.210
AV	0.000	0.003	0.513	0.150	0.144	0.106	0.003	0.602	0.177	0.173	0.574	0.167	0.162	0.127	0.413	0.455	0.805	0.016	0.327	0.335
AFV	0.159	0.101	0.693	0.127	0.175	0.271	0.054	0.701	0.022	0.430	0.673	0.028	0.327	0.441	0.652	1.048	1.175	0.361	0.477	0.117
DAV	0.100	0.196	0.015	0.131	0.382	0.354	0.150	0.102	0.065	0.399	0.183	0.066	0.195	0.152	0.128	0.340	0.315	0.258	0.221	0.058
DV/TV	0.232	0.236	0.587	0.251	0.283	0.139	0.045	0.361	0.038	0.037	0.247	0.011	0.018	0.162	0.291	0.349	0.572	0.007	0.169	0.194
AV/TV	0.085	0.166	0.110	0.074	0.293	0.082	0.060	0.190	0.159	0.364	0.296	0.264	0.521	0.279	0.043	0.185	0.038	0.240	0.007	0.242
AFV/TV	0.046	0.060	0.290	0.046	0.044	0.075	0.106	0.229	0.005	0.101	0.368	0.113	0.033	0.011	0.261	0.430	0.405	0.108	0.132	0.050
DAV/TV	0.156	0.309	0.242	0.192	0.319	0.218	0.145	0.113	0.034	0.125	0.004	0.111	0.046	0.118	0.086	0.031	0.088	0.086	0.005	0.090
BL	0.004	0.150	0.162	0.075	0.109	0.065	0.149	0.169	0.082	0.106	0.248	0.207	0.068	0.085	0.112	0.246	0.192	0.187	0.130	0.030
TAL	0.595	0.292	0.265	0.264	0.230	0.113	0.029	0.416	0.565	0.656	0.157	0.194	0.223	0.317	0.048	0.105	0.299	0.068	0.305	0.241
AFL	0.890	0.555	0.553	0.340	0.339	0.257	0.352	0.608	0.762	0.832	0.132	0.322	0.358	0.498	0.259	0.301	0.478	0.022	0.158	0.144
AL	0.016	0.052	0.056	0.101	0.120	0.134	0.307	0.426	0.758	1.013	0.018	0.359	0.530	0.664	0.436	0.669	0.852	0.145	0.276	0.141
DL	0.349	0.079	0.008	0.026	0.250	0.363	0.348	0.359	0.383	0.726	0.073	0.061	0.414	0.590	0.018	0.274	0.396	0.329	0.478	0.099
TRL	1.079	0.982	0.813	0.615	0.252	0.296	0.011	0.231	0.324	0.956	0.201	0.290	0.856	0.977	0.115	0.659	0.753	0.455	0.509	0.010
HL	0.152	0.275	0.109	0.290	0.149	0.323	0.490	0.303	0.481	0.334	0.201	0.051	0.129	0.018	0.227	0.056	0.249	0.162	0.042	0.163
HB	0.987	0.406	0.272	0.267	0.130	0.106	0.525	0.677	0.556	0.695	0.134	0.087	0.218	0.333	0.031	0.103	0.189	0.118	0.196	0.050

SL	0.516	0.673	0.429	0.550	0.372	0.359	0.219	0.082	0.181	0.168	0.063	0.013	0.375	0.477	0.062	0.190	0.244	0.301	0.372	0.061
ML	0.737	0.914	0.663	0.530	0.395	0.701	0.411	0.161	0.080	0.251	0.174	0.203	0.539	0.455	0.046	0.317	0.199	0.228	0.112	0.211
ED	0.763	0.835	0.590	0.448	0.243	0.294	0.159	0.040	0.014	0.569	0.170	0.082	0.669	0.664	0.037	0.423	0.409	0.330	0.318	0.042
EI	1.139	1.138	0.383	0.664	0.591	0.293	0.287	0.466	0.474	0.529	0.342	0.301	0.373	1.020	0.109	0.061	0.229	0.062	0.494	0.412
HLE	0.469	0.816	0.511	0.454	0.338	0.433	0.470	0.062	0.077	0.076	0.405	0.311	0.465	0.690	0.026	0.126	0.220	0.129	0.203	0.029
BD	0.318	0.693	0.236	0.329	0.254	0.227	0.282	0.077	0.026	0.066	0.375	0.237	0.367	0.513	0.100	0.013	0.052	0.089	0.164	0.068
BB	0.658	0.661	0.396	0.534	0.252	0.250	0.046	0.292	0.205	0.389	0.265	0.169	0.370	0.472	0.112	0.121	0.184	0.233	0.320	0.042
PL	0.746	0.772	0.454	0.626	0.346	0.142	0.05	0.269	0.168	0.83	0.248	0.133	0.851	0.791	0.124	0.638	0.406	0.757	0.610	0.475

Table S4. Euclidean distances of seven *C. myriaster* populations.

Population	QD	RZ	RS	WH	ZS	LYG	DL
QD	0						
RZ	6.760	0					
RS	4.974	6.992	0				
WH	9.203	12.759	6.836	0			
ZS	12.139	6.496	12.565	17.710	0		
LYG	10.740	6.131	11.295	16.010	3.261	0	
DL	13.274	13.100	14.892	17.604	13.759	13.139	0

Table S5. The accuracy of discriminant function analyses in each population.

**Population**		QD	RZ	RS	WH	ZS	LYG	DL	Total
**Quantity**		41	25	29	21	30	32	40	218
**Accuracy (%)**		68.29	36.00	48.28	61.91	60.00	59.38	72.50	59.63
**Predictive classification results**	QD	28	7	3	2	0	1	0	41
	RZ	2	9	4	5	1	0	4	25
	RS	2	7	14	6	0	0	0	29
	WH	2	2	4	13	0	0	0	21
	ZS	1	1	0	0	18	6	4	30
	LYG	0	0	0	0	11	19	2	32
	DL	1	2	0	0	7	1	29	40
